# Infiltrating Lipoma of the Right Ventricle Involving the Interventricular Septum and Tricuspid Valve: Report of a Rare Case and Literature Review

**DOI:** 10.1097/MD.0000000000002561

**Published:** 2016-01-22

**Authors:** Lingyun Fang, Lin He, Yan Chen, Mingxing Xie, Jing Wang

**Affiliations:** From the Department of Ultrasound (LF, LH, MX, JW); and Department of Radiology (YC), Union Hospital, Tongji Medical College, Huazhong University of Science and Technology, Wuhan, China.

## Abstract

Cardiac lipoma, which are primary cardiac tumors, are rare entities often detected incidentally during imaging. There have been very few reports on the right ventricle (RV) lipoma. Here, we present a case of RV infiltrating lipoma involving the interventricular septum (IVS) and the tricuspid valve. Clinical symptoms, diagnostic procedures, multimodality imaging characteristics, and treatment are discussed, and the complete clinical data of this case and relevant details of retrospective literature are reviewed. The study described the case of a 48-year-old woman who suffered from occasional palpitation after exertion for 10 years. Imaging examinations, including echocardiography and cardiovascular magnetic resonance imaging (MRI), revealed a large mass adherent to the IVS and the right ventricular wall that was consistent with lipoma. The patient underwent surgical repair of the tricuspid valve and excision of the partial mass. The gross specimen revealed piles of 5 × 4 × 3 cm fragments with yellowish appearance and pathological results showed infiltrating lipoma.

Lipoma is often asymptomatic and diagnosed incidentally. Surgical excision is the main therapeutic intervention, which is always performed in cases of symptomatic lipoma or when malignancy is suspected. Multimodality imaging would be great help in the diagnosis of cardiac lipoma. Echocardiography is a convenient method for follow-up.

## INTRODUCTION

Lipoma is a rare primary cardiac tumor that is often asymptomatic and diagnosed incidentally. It can be found throughout the heart, typically in subepicardial or subendocardial locations. Rarely, they arise within the myocardium or from the valve leaflets. Clinical manifestations vary and depend on the location and size of the mass.^1^ In our case, the patient had atypical symptoms for 10 years, and only isolated premature ventricular beats were recorded. Tumors infiltrating the ventricular wall might damage the myocardium or conduction system and result in ventricular arrhythmias.^2^ Although cardiac lipoma is uncommon, they are easily diagnosed by the imaging examination and should be confirmed by the pathological examination. Surgical resection is necessary for symptomatic cardiac lipoma. Few reports have described the details of right ventricle (RV) lipoma. We sought to share our experience with a rare case of RV infiltrating lipoma. Complete data about the multimodality imaging examinations, including 2-dimensional transthoracic echocardiography (2D-TTE), real-time 3-dimensional echocardiogram (RT-3DE), contrast-enhanced ultrasound (CEUS) and MRI, and surgical and pathological findings were recorded and discussed. The study was approved by the local research ethics committee at Union hospital, Tongji medical college, Huazhong University of Science and Technology, China. The individual in this manuscript has given written informed consent to publish these case details. In addition, we reviewed RV lipoma-related literature from the past 20 years in order to provide useful information about the diagnosis of this condition.

## CASE REPORT

### Case History and Physical Examination

A 48-year-old woman presented having experienced occasional palpitation on exertion for 10 years that had worsened during the past 6 months. Two weeks earlier, she was found to have a cardiac mass at a local hospital. The patient was then referred to our hospital to receive further evaluation and management in February 2015. Her medical history and family history were unremarkable. She presented with weight loss and vital signs of: pulse rate, 76 beats/min; blood pressure, 113/65 mm Hg; and respiratory rate, 20 breaths/min. The results of the physical examination were normal and there were no specific physical signs of the heart. Laboratory tests demonstrated normal liver function, the white blood cell count was 10 060/mm^3^, and ECG showed normal sinus rhythm with isolated premature ventricular beats from the RV. The chest x-ray was normal. An echocardiogram was performed for the evaluation of cardiac structure and function.

### Echocardiographic Examination

**FIGURE 1:**
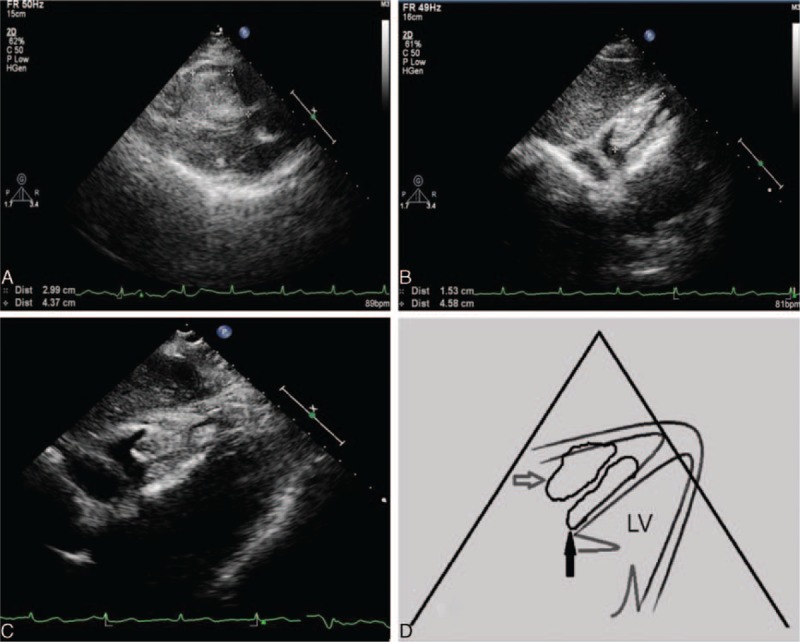
Echocardiographic findings show a large mass involving the IVS (A) and the RV wall (B) lining toward the RV cavity. The subxiphoid 4-chamber view displays the mass adherent to the IVS (white arrow) and the RV wall (black arrow; C). Schematic diagram of the subxiphoid 4-chamber view (D). IVS = interventricular septum; RV = right ventricle.

**FIGURE 2:**
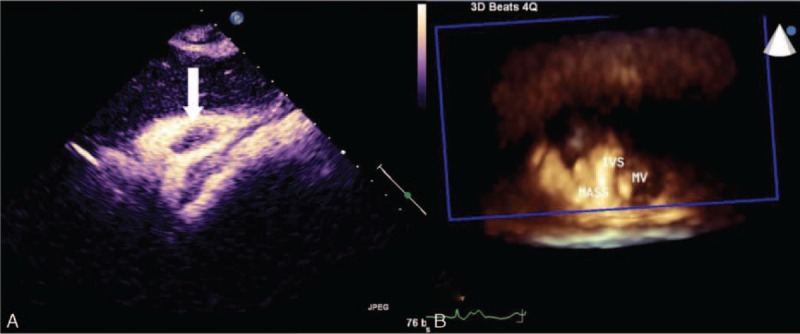
Contrast enhancement ultrasound findings show few contrast agent enhancements in the tumor structure (white arrow; A); real-time 3D full-volume bird's eye view findings of the mass adherent to the IVS and attached to the RV wall (B). IVS = interventricular septum; MV = bicuspid valve; RV = right ventricle.

2D-TTE (Philips IE33; Philips Healthcare, Eindhoven, Netherlands) demonstrated an irregular hyperechoic mass adherent to the IVS with a broad base (Figure 1A). The size of the mass was 4.4 × 3.0 × 2.4 cm^3^ and the left ventricular ejection fraction was 67%. Incidentally, another echogenic mass measuring 4.6 × 1.5 cm was observed to be adherent to the RV wall (Figure 1B); it was irregular and showed good mobility. In the subxiphoid 4-chamber view, the mass was adherent to the IVS and the RV wall (Figure 1C and D). Color Doppler flow imaging (CDFI) did not detect any flow signal inside the mass. The flow of the RV outflow tract and the tricuspid orifice were not obstructed and mild tricuspid regurgitation was detected in systole. CEUS demonstrated that there was a slight enhancement of the contrast agent inside the mass (Figure 2A). However, subsequent RT-3DE showed the involved scope of the mass was nearly attached to the right ventricular apex (Figure 2B). In conclusion, the diagnosis was an irregular RV mass located in the right side of the IVS and the RV wall, which was probably connected.

### Magnetic Resonance Imaging

**FIGURE 3:**
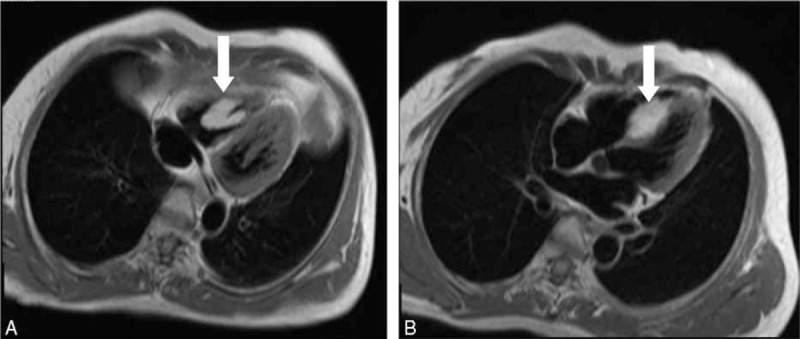
An echogenic RV mass is seen on transthoracic echocardiography. The horizontal, long-axis (4-chamber view), T1-weighted, black-blood image showing a well-circumscribed, high-signal mass in the RV cavity (arrow, A). The horizontal, long-axis, T1-weighted black-blood image showing the mass adherent to IVS (arrow, B). IVS = interventricular septum; LA = left atrium; LV = left ventricle; RA = right atrium; RV = right ventricle.

To further characterize the mass, the patient underwent cardiovascular MRI. Images revealed a homogeneous high-signal intensity within a quite irregular mass, which was adherent to the RV side of the IVS with a broad base that measured approximately 4.0 × 1.6 cm in maximal transverse diameter (Figure 3). The mass extended to the RV wall. The signal characteristics of the mass were similar to those of the thoracic muscle tissue signal on true-fast imaging with steady-state free precession (TRUFI) sequence images; thus, the lesion was suspected to be a lipoma.

### Surgical and Pathological Findings

**FIGURE 4:**
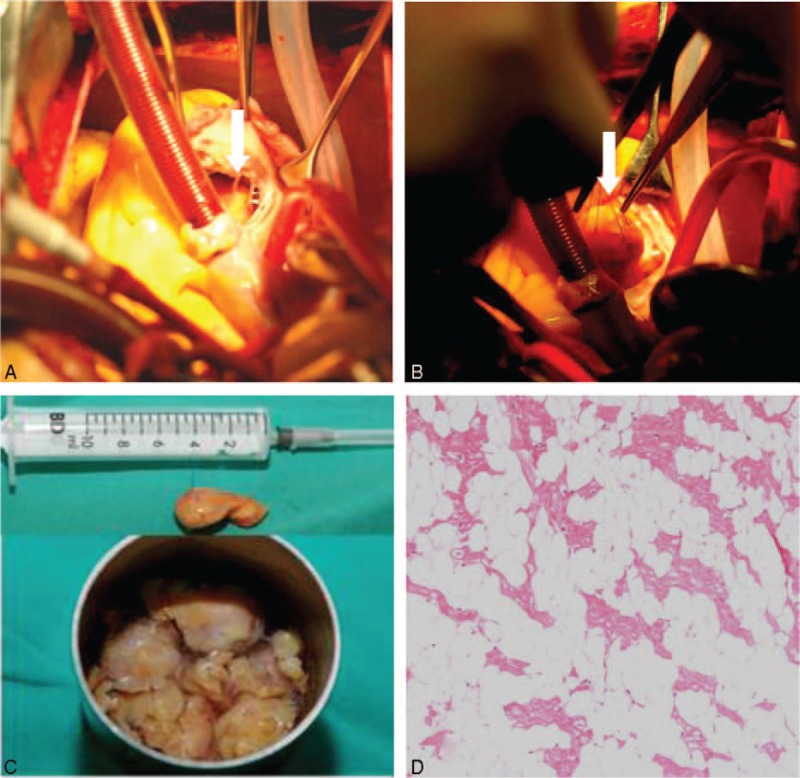
Surgical and pathology findings show a yellowish tumor involving the chordae of tricuspid valve (arrow, A); the tumor is suspended by sutures (arrow, B). After resection of the tumor, a pile of tissue in the water (C); stained with hematoxylin and eosin (H&E) at 100 magnification: the tumor comprised mature adipocytes with entrapped myocardial cells (D). H&E = hematoxylin and eosin.

The patient underwent surgery to remove the mass, under the diagnosis of RV tumor. During surgery, an irregular, yellowish, soft mass was found to be embedded in the IVS, and to occupy the RV cavity and the right ventricular apex. The tumor involved the chordae of the tricuspid septal and posterior leaflets. The tumor in the right ventricular apex was completely resected, but it was impossible to remove the entire tumor, because it was integrated with and partly immersed in the IVS. The tumor was removed in pieces, with part of the IVS and the tricuspid valve chordae (Figure 4A and B). Tricuspid valve repair was performed by construction of new chordae from the autologous pericardium. The reconstruction was performed with a clipped pericardium into the chordae, with one end attached to the appropriate papillary muscle and the other attached to the valve leaflet. The aim was to suspend the leaflet to prevent tricuspid regurgitation. Water was injected into the right ventricle to test the competence of the valve. The gross appearance of the surgically resected tumor was a pile of yellowish pieces of smooth tissue (Figure 4C). A microphotograph showed that the mass was composed of a cluster of mature adipocytes with entrapped myocardial cells. Removed tissue sections showed a massive infiltration of mature adipocytes with displacement of many pre-existing myocardial cells. Histological examination of the surgical specimen was used to determine the diagnosis of infiltrating lipoma of the RV (Figure 4D).

### Follow-Up

On the seventh postoperative day, echocardiography revealed that a remarkably reduced tumor of 2.9 × 1.1 cm remained in the IVS, but the tumor located in the RV wall was completely absent. An interesting finding was the detection of severe regurgitation of the tricuspid valve. The patient was asymptomatic at 1 and 3 months after surgery, and her follow-up echocardiography showed normal left ventricular ejection fraction. The tricuspid regurgitation was dramatically improved and mild physiologic tricuspid regurgitation was detected.

## DISCUSSION

### Epidemiology and Pathology

Primary cardiac tumors are a group of rare disorders with a frequency that varies between 0.0017% and 0.02% in population studies.^3^ The lipoma is a relatively rare cardiac tumor, accounting for 8.4% of benign primary cardiac tumors, with a broad range of appearance and sizes.^4^ Another review reported the incidence of cardiac lipoma among benign primary cardiac tumors was 2.4%.^5^ These differences in incidence may be related to the inherent bias of different research institutes. Lipomas differ by neither age nor gender.^6^ Cardiac lipoma includes lipoma, lipomatous hypertrophy of the interatrial septum (LHIS), and intermuscular lipoma (also known as lipomatous infiltration).^7^ Lipomas comprise an encapsulated mass of mature adipocytes and should be clearly distinguished from the LHIS. The latter entity is the local hyperplasia of mature adipocytes and/or embryonic adipose tissue, which are only located in the interatrial septum resulting in interatrial septum hypertrophy. LHIS is not a genuine tumor and is more common than cardiac lipoma, it is more frequent in elderly, obese patients, and is usually an incidental finding during a variety of cardiac imaging or surgical procedures.^8^ Lipoma invaded the myocardial layer, which is termed infiltrating lipoma, may cause conduction disturbances, arrhythmia, and sudden death.^9^ The present case was of lipoma attached to the IVS with a broadbase, and the patient had an abnormal electrocardiogram consistent with infiltrating lipoma by pathology. Although lipoma may occur throughout the heart, it is most frequently located on the left side of the heart^10^ and is often found in the interventricular septum and valves (valve “fibrolipoma”).^11^ Lipomas can originate from all 3 layers of cardiac tissue, the subendocardium (50%), subpericardium (25%), and myocardium (25%), with a predilection for the visceral and parietal pericardium.^11^ Chiefly, they arise within the myocardium or from the valve leaflets.^12^ One review reported that the variation in location of cardiac lipoma.^13^ Related articles on PubMed from 1995 to 2014 were searched using the terms of “cardiac tumor, lipoma” in English. We retrospectively analyzed the location of cardiac lipoma. A total of 208 cases of cardiac lipoma were identified, which occurred in any position in the heart (Table 1). Interatrial septum was the most common position of the cardiac lipoma (80/208 cases, 38.5%), and 57 cases were LHIS. Lipomas have a special predilection for the right atrium (37/208 cases, 17.8%) and were more common in the pericardium (27/208 cases, 13.0%) and left ventricle (24/208 cases, 11.5%). Of all cases of cardiac lipoma, only 10 tumors were found in the right ventricle (4.8%), of these, 6 patients were from case reports and the other 4 cases were reported respectively in 2 large-scale retrospective studies.^14–21^ These articles were selected for review and are summarized below in the discussion of the literature (Table 2). In the present case study, during surgery, a lipoma was identified on the apex of the RV and the right side of IVS, which involved the chordae of the tricuspid valve, and the location was consistent only with the findings of Nishi.^16^ There were 90% of cases underwent surgery and histological results showed 4 cases, including ours, of infiltrating lipoma. One patient was asymptomatic and the case was managed conservatively.

**TABLE 1:**
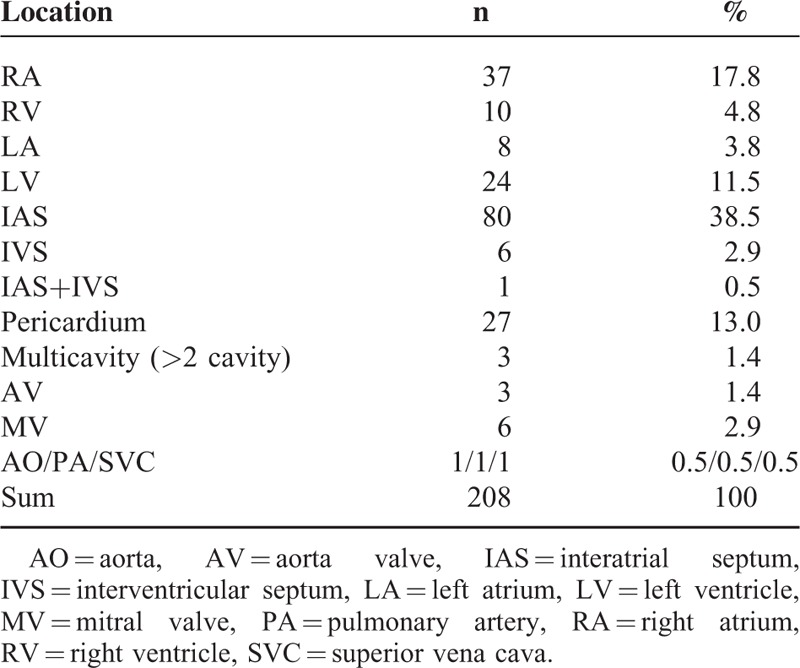
Literature Review of Cardiac Lipoma Location

**TABLE 2:**
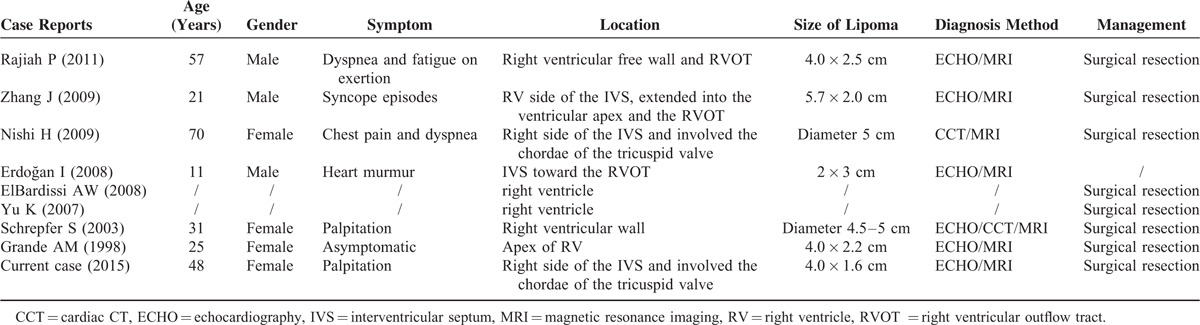
Literature Review of Right Ventricular Cardiac Lipoma Case Reports

### Clinical Symptoms of Cardiac Lipoma

With respect to primary cardiac tumors, clinical presentation varies depending on the site and size of the lipoma, and many are identified incidentally at autopsy or during cardiac imaging investigation. Table 2 shows a summary of cases of RV lipoma that were described in our case report and in the literature. Symptoms include dyspnea, palpitation, and chest pain. These symptoms may be ambiguous or significant. Conduction disturbances caused by myocardium invasion would lead to arrhythmia, such as premature beats or atrial fibrillation. In our case, the patient had developed worsening palpitation, which might have been related to premature beats. Giant lipoma located in the cardiac chambers or pericardium may be caused by tamponade symptoms or valve dysfunction. Fortunately, in our case, the tricuspid regurgitation was mild because the growth of the mass was unobstructed by the tricuspid orifice and the mobility of tricuspid valve was intact. Furthermore, unlike myxoma, cardiac lipoma is more stable and was uncommon for pulmonary or peripheral embolization.^3^

### Echocardiographic Approach to the Diagnosis of Cardiac Lipoma

Echocardiography is the most suggestive noninvasive method that could provide adequate accurate information about the cardiac mass. Two-dimensional echocardiography, and more recently RT-3DE,^22^ can display morphological characteristics of lipoma, including tumor texture, size, shape, location, attachment, mobility, and other secondary hemodynamic changes. RT-3DE could provide vivid data regarding the mass and its adjacent structures, which are benefit for interactions with the surgeon and management. CDFI may be useful for evaluating the hemodynamic consequences of valvular dysfunction or obstructive changes caused by cardiac tumors. Transesophageal echocardiography (TEE), which should always be considered when the transthoracic study is suboptimal or confusing,^23^ has the advantage of being able to detect small masses or unclear cardiac structure. CEUS may describe tumor vascularity. Typically, cardiac lipoma may have fewer vessels, and a rich vascularization may suggest a malignancy. Cardiac lipoma is homogeneous without calcifications and has a markedly hyper-reflective mass and adipose tissue. It tends to be of irregular shape and without pedicle. The echocardiographic features of lipomas are fairly nonspecific, but they have been described to be immobile and well circumscribed, with a bright homogenous appearance.^24^ Differential diagnosis of an RV mass includes thrombus, vegetation, and tumor, as well as normal nonpathologic structures that may be mistaken for masses. RV thrombi are uncommon and are usually associated with RV myocardial infarction or RV cardiomyopathy. Because right-sided cardiac vegetation would be rare, usually located in tricuspid valve, endocarditis of the right heart is common in intravenous drug abuse.^25^ Tumors have differential diagnoses, including both primary and metastatic tumors, and right atrial tumors predominate over RV tumors.^26^ Patients with metastatic tumors usually suffer from primary disease. Myxomas represent the most common neoplasms among primary benign cardiac tumors, and RV myxoma may manifest as single or multiple intracardiac homogeneous, polypoid, or papillary masses attached to the endocardium by a pedicle or with a broadbase. However, their size can vary from <1 cm to filling the whole cavity, and the mobility of myxoma depends on its size and the type of attachment.^27^

### Comparison of Different Diagnostics Imaging Techniques Methods

Noninvasive imaging technique methods play a critical role in the diagnosis and surgical management of cardiac tumors. Echocardiography is the first choice method for assessment of cardiac tumors because of its widespread availability, portability, or lack of radiation exposure. The advantage of echocardiography examination is the real-time assessment of the morphological features of cardiac tumors and hemodynamic changes. The technique is convenient and accurate for cardiac function assessment and follow-up. Particularly, intraoperative TEE is indicated for the evaluation of resection of cardiac tumors, and may guide surgical resection. Assessment of any hemodynamic consequence is also an important role of the prebypass TEE examination.^28^ In addition, it is necessary for TEE-guided transvenous biopsy, which is a safe and effective method of obtaining tissue for histologic diagnosis in right-sided cardiac masses.^26^ However, echocardiography provides limited assessment of soft-tissue characteristics and extracardiac structures and may be limited by poor acoustic windows.^13^

Cardiac CT has high temporal and spatial resolution, fast scanning imaging and reconstruction capabilities for assessment the cardiac tumor, especially when other imaging techniques have contraindications or unavailable, when patients have contraindications to MRI, cardiac CT provides an alternative imaging method.^13^ It is beneficial to offer more diagnosis information about cardiac lipoma and display fine intracardiac or extracardiac structures which are restricted view by echocardiography. Cardiac lipoma have low attenuation characteristic and a homogenous appearance of fat tissue on cardiac CT, measurement the Hounsfield Units (HU) in the lesion area, the density values may be < −50 HU.^13,29^

Cardiac MRI provides extensive visual fields, multiplanar imaging without reconstruction, especially, the excellent soft-tissue contrast is helpful for tissue characterization and management cardiac lipoma.^30^ It can display the location of the masses and the involved area accurately, and show the surroundings relationship of cardiac masses. MRI is a powerful diagnostic method for cardiac lipoma, which has a high-signal on T1-weighted sequences, but should differential with thrombi.^29^ The multimodality imaging techniques play an integrated role to obtain the maximize advantages for assessment and diagnosis cardiac lipoma accurately.^31^

### Management of Cardiac Lipoma

Lipomas are often asymptomatic and incidentally discovered on echocardiography, such as in cardiac CT or MRI. They may cause arrhythmias, conduction system disturbances, symptoms of heart failure, and so on, especially in cases where they reach a large size.^32^ Large lipomas, particularly when associated with severe symptoms, should be resected.^33^ Smaller, asymptomatic lipomas, should be observed clinically.^17^ In our case, the patient underwent partial resection of the tumor and tricuspid valve repair, in view of the involvement of the IVS and the chordae of tricuspid. On the seventh postoperative day, follow-up echocardiography revealed severe tricuspid regurgitation. At 1 and 3-month follow-ups, TTE showed significant improvement of tricuspid regurgitation. The reason might be related to myocardial reperfusion injury and operative injury resulting in papillary muscle dysfunction, decreased cardiac function, annular dilation, and so on, which would be restored with longer follow-up.

## CONCLUSION

Cardiac lipoma is an uncommon type of primary cardiac tumor, and RV lipoma is extremely rare. There is no defined age or sex distribution and it can present with a wide range of symptoms. Echocardiography remains the initial diagnostic tool for cardiac lipoma. However, cardiac CT and MRI provide better image resolution and details before surgical resection. They are often used synergistically with echocardiography in the evaluation and management of cardiac masses. Surgical resection remains the mainstay of treatment of symptomatic cardiac lipoma.

## References

[1] Miralles A Bracamonte L Souncul H Cardiac tumors:clinical experience and surgical results in 74 patients. *Ann Thorac Surg* 1991; 52:886–895. 1929651 10.1016/0003-4975(91)91241-m

[2] Kusano KF Ohe T Cardiac tumors that cause arrhythmias. *Card Electrophys Rev* 2002; 6:174–177. 10.1023/a:1017936622990 11984043

[3] Silverman N Primary cardiac tumors. *Ann Surg* 1980; 191:127–138. 7362282 10.1097/00000658-198002000-00001 PMC1345598

[4] Reece IJ Cooly DA Frazier OH Cardiac tumors: clinical spectrum and prognosis of lesions other than classical benign myxoma in 20 patients. *J Thorac Cardiovasc Surg* 1984; 88:439–446. 6381889

[5] Arı H Arı S Göncü MT Biventricular lipoma (first case in literature). *Int J Cardiol* 2011; 150:e98–e100. 20557958 10.1016/j.ijcard.2010.02.058

[6] Akram K Hill C Neelagaru N A left ventricular lipoma presenting as heart failure in a septuagenarian: a first case report. *Int J Cardiol* 2007; 114:386–387. 16624434 10.1016/j.ijcard.2005.11.083

[7] Li FP Wang XF Xiao J Myocardial lipomatous infiltration of the left ventricular wall. *J Card Surg* 2010; 25:513–515. 20331480 10.1111/j.1540-8191.2010.01021.x

[8] O’Connor S Recavarren R Nichols LC Lipomatous hypertrophy of the interatrial septum: an overview. *Arch Pathol Lab Med* 2006; 130:397–399. 16519573 10.5858/2006-130-397-LHOTIS

[9] Burke AP Litovsky S Virmani R Lipomatous hypertrophy of the atrial septum presenting as a right atrial mass. *Am J Surg Pathol* 1996; 20:678–685. 8651346 10.1097/00000478-199606000-00004

[10] Benvenuti LA Mansur AJ Lopes DO Primary lipomatous tumors of the cardiac valves. *South Med J* 1996; 89:1018–1020. 8865802 10.1097/00007611-199610000-00020

[11] Puvaneswary M Edwards JR Bastian BC Pericardial lipoma: ultrasound, computed tomography and magnetic resonance imaging findings. *Aust Radiol* 2000; 44:321–324. 10.1046/j.1440-1673.2000.00821.x 10974728

[12] Dias RR Fernandes F Ramires FJ Mortality and embolic potential of cardiac tumors. *Arq Bras Cardiol* 2014; 103:13–18. 25029470 10.5935/abc.20140096 PMC4126756

[13] Kassop D Donovan MS Cheezum MK Masses on cardiac CT: A review. *Curr Cardiovasc Imaging Rep* 2014; 7:9281. 25018846 10.1007/s12410-014-9281-1 PMC4090749

[14] Rajiah P To AC Tan CD Multimodality imaging of an unusual case of right ventricular lipoma. *Circulation* 2011; 124:1897–1898. 22025640 10.1161/CIRCULATIONAHA.111.030213

[15] Zhang J Chong E Chai P Contrasting fatty involvement of the right ventricle: lipoma versus lipomatous hypertrophy. *Singapore Med J* 2009; 50:e342–e345. 19907871

[16] Nishi H Mitsuno M Ryomoto M Giant cardiac lipoma in the ventricular septum involving the tricuspid valve. *Ann Thorac Surg* 2009; 88:1337–1339. 19766837 10.1016/j.athoracsur.2009.02.052

[17] Erdoğan I Alehan D Hazirolan T Right ventricular lipoma. *Anadolu Kardiyol Derg* 2008; 8:E25–E26. 18676291

[18] ElBardissi AW Dearani JA Daly RC Survival after resection of primary cardiac tumors: a 48-year experience. *Circulation* 2008; 118 14 Suppl:S7–S15. 18824772 10.1161/CIRCULATIONAHA.107.783126

[19] Yu K Liu Y Wang H Epidemiological and pathological characteristics of cardiac tumors: a clinical study of 242 cases. *Interact Cardiovasc Thorac Surg* 2007; 6:636–639. 17670730 10.1510/icvts.2007.156554

[20] Schrepfer S Deuse T Detter C Successful resection of a symptomatic right ventricular lipoma. *Ann Thorac Surg* 2003; 76:1305–1307. 14530040 10.1016/s0003-4975(03)00523-x

[21] Grande AM Minzioni G Pederzolli C Cardiac lipomas. Description of 3 cases. *J Cardiovasc Surg* 1998; 39:813–815. 9972906

[22] Asch FM Bieganski SP Panza JA Real-time 3-dimensional echocardiography evaluation of intracardiac masses. *Echocardiography* 2006; 23:118–124. 10.1111/j.1540-8175.2006.00196.x 16524392

[23] Tan CN Fraser AG Transesophageal echocardiography and cardiovascular sources of embolism: implications for perioperative management. *Anesthesiology* 2007; 107:333–346. 17667579 10.1097/01.anes.0000270733.26234.56

[24] Mehta RK Navin NC Osman K Left ventricular lipoma by transesophageal and in vitro echocardiographic studies. *Echocardiography* 1995; 12:283–288.

[25] Elsevier Saunders Publishing, Catherine MO Rebecca GS Rosario VF Echocardiography Review Guide: Companion to the Textbook of Clinical Echocardiography. 2nd 2011; 299–321.

[26] Lynch M Clements SD Shanewise JS Right-sided cardiac tumors detected by transesophageal echocardiography and its usefulness in differentiating the benign from the malignant ones. *Am J Cardiol* 1997; 79:781–784. 9070559 10.1016/s0002-9149(96)00868-5

[27] Humana Press, Basso C Valente M Thiene G Badano LP Muraru D Lliceto S Cardiac tumor pathology. *Echocardiography of Cardiac Masses: From Two- to Three-Dimensional Imaging* 2013; 101–114.

[28] Tanzola RC Allard R Hamilton A Intraoperative transesophageal echocardiography for an intracavitary left ventricular lipoma. *Anesth Analg* 2009; 108:786–787. 19224783 10.1213/ane.0b013e3181938890

[29] Salanitri JC Pereles FS Cardiac lipoma and lipomatous hypertrophy of the interatrial septum cardiac magnetic resonance imaging findings. *J Comput Assist Tomogr* 2004; 28:852–856. 15538164 10.1097/00004728-200411000-00022

[30] Sparrow PJ Kurian JB Jones TR MR imaging of cardiac tumors. *Radiographics* 2005; 25:1255–1276. 16160110 10.1148/rg.255045721

[31] Krombach GA Spuentrup E Buecker A Heart tumors: magnetic resonance imaging and multislice spiral CT. *Rofo* 2005; 177:1205–1218. 16123866 10.1055/s-2005-858489

[32] Verberkmoes NJ Kats S Tan-Go I Resection of a lipomatous hypertrophic interatrial septum involving the right ventricle. *Interact Cardiovasc Thorac Surg* 2007; 6:654–657. 17670727 10.1510/icvts.2007.157776

[33] Zhu SB Zhu J Liu Y Surgical treatment of a giant symptomatic cardiac lipoma. *J Thorac Oncol* 2013; 8:1341–1342. 24457246 10.1097/JTO.0b013e3182a12a6a

